# An ingenious non-spherical mesoporous silica nanoparticle cargo with curcumin induces mitochondria-mediated apoptosis in breast cancer (MCF-7) cells

**DOI:** 10.18632/oncotarget.26623

**Published:** 2019-02-05

**Authors:** Lakshminarasimhan Harini, Sweta Srivastava, George Peter Gnanakumar, Bose Karthikeyan, Cecil Ross, Vaithilingam Krishnakumar, Velu Rajesh Kannan, Krishnan Sundar, Thandavarayan Kathiresan

**Affiliations:** ^1^ Department of Biotechnology, Kalasalingam University, Krishnankoil, Tamil Nadu, India; ^2^ Department of Translation Medicine, St. Johns National Academy of Health Sciences, Bangalore, Karnataka, India; ^3^ School of Physical Chemistry, Madurai Kamaraj University, Madurai, Tamil Nadu, India; ^4^ Oregon Health and Science University, Knight Cardiovascular Institute (KCVI), Portland, Oregon, USA; ^5^ Department of Medicine, St. Johns National Academy of Health Sciences, Bangalore, Karnataka, India; ^6^ Department of Microbiology, Bharathidasan University, Tiruchirappalli, Tamil Nadu, India; ^7^ International Research Centre, Kalasalingam University, Krishnankoil, Tamil Nadu, India

**Keywords:** caspase, mesoporous silica nanoparticle, toxicity, mitochondria, doxorubicin

## Abstract

Curcumin delivery to cancer cells is challenging due to its hydrophobic nature, low bio distribution and low availability. Many nano vehicles suffer from low stability and toxicity, and hence the prerequisite of a non-toxic nano vehicle with effective drug delivery is still being delved. The present study investigates the delivery efficiency of curcumin with non-spherical mesoporous silica nanoparticles (MSNAs). Their mechanism of drug delivery and signalling proteins activated to induce apoptosis was further explored in MCF-7 cells. A non-spherical MSN was synthesised, functionalised with PEI (MSNAP) and analysed its intracellular behaviour. Our result indicates that MSNAP was non-toxic until 20 µg/mL and likely localizes in cytoplasmic vesicles. On contrast, well-known MCM-41P induced autophagosome formation, indicating cellular toxicity. Curcumin was loaded on MSNAP and its effectiveness in inducing cell death was studied in MCF-7 and in MCF-7R cells. Curcumin loading on MSNAP induces better cell death with 30 µM curcumin, better than unbounded curcumin. Western blot analysis suggest, curcumin induce apoptosis through the activation of caspase 9, 6, 12, PARP, CHOP and PTEN. The cell survival protein Akt1 was downregulated by curcumin with and without the nanostructure. Interestingly, cleaved caspase 9 was activated in higher amount in nano-conjugated curcumin compared to the free curcumin. But other ER resident protein like IRE1α, PERK and GRP78 were downregulated indicating curcumin disturbs ER homeostasis. Further, electron microscopic analysis reveled that nanocurcumin induced apoptosis by disrupting mitochondria and nucleus. Our results with doxorubicin resistant MCF-7 cell lines confirm nanodelivery of doxorubicin and curcumin sensitised cells effectively at lesser concentration. Further docking studies of curcumin indicate it interacts with the apoptotic proteins through hydrogen bonding formation and with higher binding energy.

## INTRODUCTION

Mesoporous silica nanoparticles have attained importance in biomedical research as a prominent drug delivery system (DDS). MSN’s more flexibility in designing, fabricating and site-specific targeting enables enhanced encapsulation of the drug [1]. Moreover, its biocompatibility, alterable porosity, controlled drug release; high cargo loading and stability emphasize the prominence of MSN in drug delivery research [2]. Surface coating of nanoparticle with polymer enhances its retention time, biocompatibility and prevents aggregation [3, 4]. Polymers like PEG, PCL, dextran, chitosan, PEI were widely used for nanoparticle sheathing, of which PEI, the cationic polymer is an efficient system for transfecting nucleic acid [5]. The ‘proton sponge’ effect of PEI aids its endosomal escape where most of the nanostructures are trapped [6]. Also PEI coating of nanostructures aids in efficient drug encapsulation and drug delivery in the cytosol [7]. PEI has efficient drug uptake and intracellular drug release but its application is limited due to cytotoxic nature.

Non-spherical nanoparticles are reported to be advantageous than the spherical nanoparticles in their compatibility, cellular uptake, biodistribution, longer circulation time, tumour accumulation, endosomal escape and tumour inhibition [8–10]. Toxicity of the nanoparticle is attributed to the induction of ROS which leads to oxidative DNA damage, membrane blabbing, protein adducts and enzyme dysfunction [11]. The high aspect ratio of long rods of MSN is less and also has reduced ROS production toxic when compared to short rods [12]. In contrary, *in vivo* study revealed that long rods are excreted less compared to the spherical particle which induced renal damage and hemorrhage [13]. Still, the effect of non-spherical MSN on cellular toxicity is debated at minimum level.

Though curcumin exhibits anticancer effect against many cancer cell lines, its poor solubility and stability fortify curcumin as the first drug of choice in nanoformulation [14]. So far, curcumin has been conjugated with liposomes, PLGA, cyclodextrin, micelles, dendrimers, polymers, metal oxides, carbon nanotubes, nanogels iron oxide and silica [15]. In spite of showing advantageous in curcumin delivery, each method had its own drawback. For instance, liposomal curcumin accumulate in liver and spleen due to low circulatory time in blood and also lack tissue specificity [16], PLGA with N-isopropylacrylamide NPs curcumin formulation encapsulate multiple particles and solid lipid nanoparticle-curcumin lacked stability and could not be stored for longer time [17].

Mitochondria and endoplasmic reticulum plays a major role in progression of cancer. Both these organelles sense cellular stress in cancer microenvironment and modify their structure and function depending on cellular demand for cancer cell survival [18]. Thus, mitochondria are considered as the prime target for an anti-cancer investigation [19]. Curcumin nanoformulation of guanidine functionalized PEGylated mesoporous silica nanoparticle was effective inducing apoptosis in human breast adenocarcinoma cells (MCF-7), and mouse breast cancer cells (4T1), but not in human mammary epithelial cells (MCF-10A) [20]. Similarly, curcumin loaded on nanoformulations like Myristic acid (MA)–Chitosan nanogel [21], amine-functionalized KIT-6, MSU-2, and MCM-41 with curcumin induces cell death [22] in MBA-MB-231 and A549 cell lines [22]. However, the detailed mechanism of nanocurcumin induced apoptosis remains elusive in cancer cells.

The present investigation elucidates PEI decorated non-spherical mesoporous silica nanoparticle (MSNAP)loaded with curcumin-induced apoptosis in both MCF-7 and MCF-7R cells. Our results indicated that MSNAP was non-toxic and accumulate rapidly intracellular in MCF-7 cells. Curcumin released from CUR-MSNAP intracellularly induced apoptosis through disturbing mitochondria and nucleus in breast cancer MCF-7 cells *in vitro*. In MCF-7R cells, DOX-MSNAP induces cell death at a lesser concentration than unbound doxorubicin. Non-toxicity, faster intracellular accumulation, and effective intracellular drug delivery signify MSNAP as better drug delivery vehicle *in vitro*.

## RESULTS

### MSNAP synthesis and biophysical characterisation

**Figure 1:**
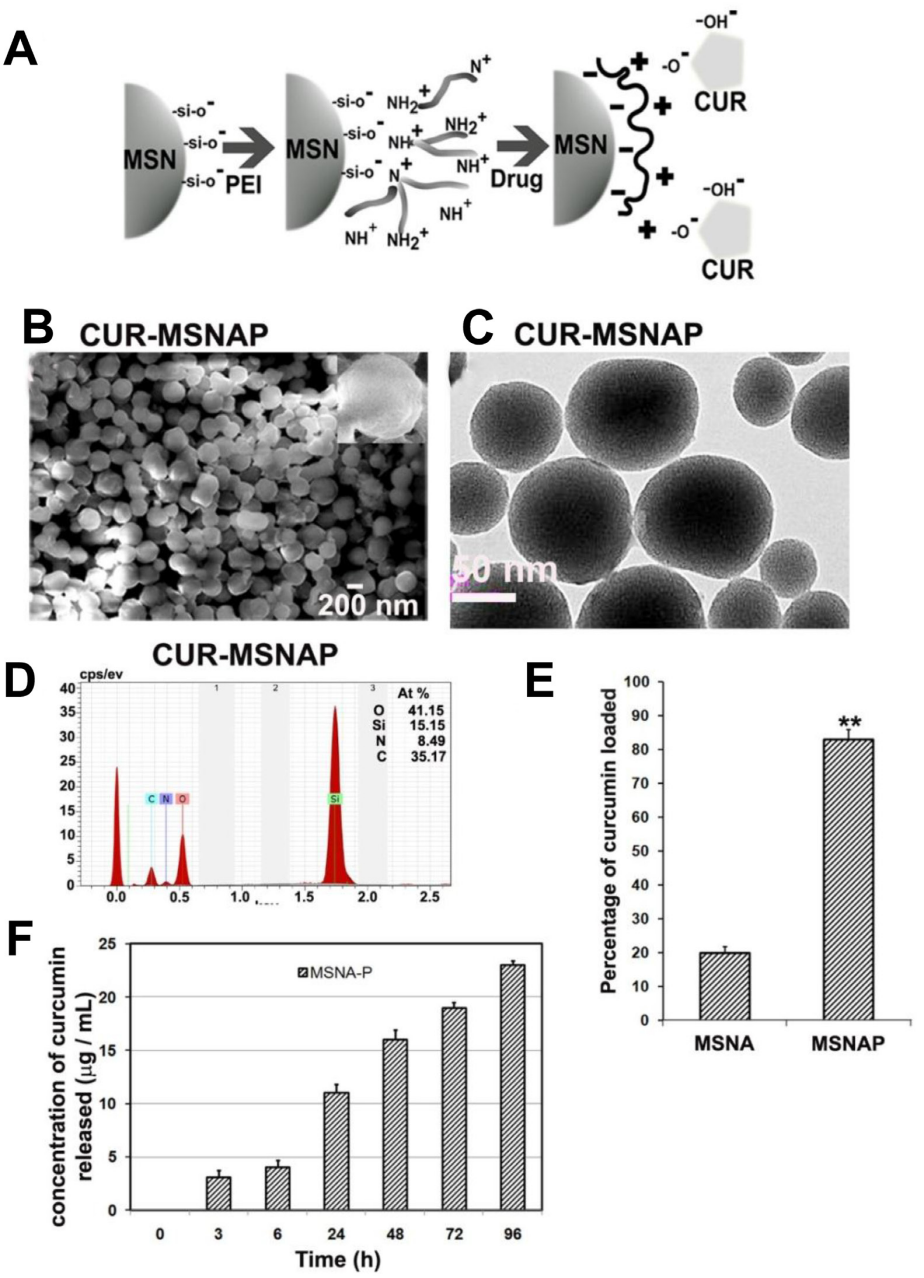
Characterization of CUR-MSNAP (**A**) Schematic representation of PEI coating and curcumin loading on MSN (**B**) 200 nm scale SEM images curcumin-loaded MSNAP, inset: single CUR-MSNAP (**C**) 50 nm scale TEM images of CUR-MSNAP (**D**) EDAX graph of CUR-MSNAP (**E**) Graph of curcumin loading percentage on MSNAP (**F**) Graph of curcumin released at different time point from CUR-MSNAP at pH 7.4. *n* = 3, ^**^ indicates *p* < 0.01 of percentage of curcumin loaded on MSNAP compared to MSNA.

MSNA was synthesized, coated with PEI and characterized for its structure and functional groups. PEI was coated on these silica particles through the electrostatic interaction between the NH^+^, NH_2_^+^ and NH_3_^+^ ions of PEI and Si-OH, Si-O-Si, O-Si-O anions of silica nanoparticles (Figure 1A). Similarly, curcumin loading on MSNAP was mediated by the electrostatic interaction between the PEI cationic groups and –OH and -C = O anionic group of curcumin. SEM image of CUR-MSNAP confirms (Figure 1B) the non-spherical shaped discoid nanostructures. The rough surface (inset) of CUR-MSNAP indicates curcumin cargo on it. Further, TEM image (Figure 1C) confirms the pores of the nanoparticle are saturated by the drug. EDAX data (Figure 1D) revealed that CUR-MSNAP was composed of Si -15.15%, O-41.15%, C–35% and N–8.49%. Presence of carbon confirms the loaded curcumin and nitrogen indicate the surface functionalization with PEI.

TEM analysis of MSNAP (data not shown), revealed the parallel arrangement of pores and variation in particle shape. TEM image of CUR-MSNAP (Figure 1C) appeared darker compared to MSNAP. Curcumin saturated the pores of MSNAP resulting in a darker image.

### Drug uptake and release by MSNAP

Drug adsorption studies were performed to determine the drug loading capacity of these nanostructures. Curcumin loading on MSNA was 20% however, PEI coated MSNA enhanced the drug loading to 80% (Figure 1E). Therefore, PEI enhanced the capacity of drug loading in MSNAP to four-fold (Figure 1E). The release of curcumin from CUR-MSNAP was monitored in PBS at pH 7.4 at various time points from 0 to 96 h (Figure 1F). A maximum of 23 µM was released from CUR-MSNAP at 96 h. In the initial ‘burst phase’ within 24 h. CUR-MSNAP released 13 µM of drug and then a sustained pattern of release was observed till 96 h.

### Toxicity evaluation of MSNAP in MCF-7 cells

**Figure 2:**
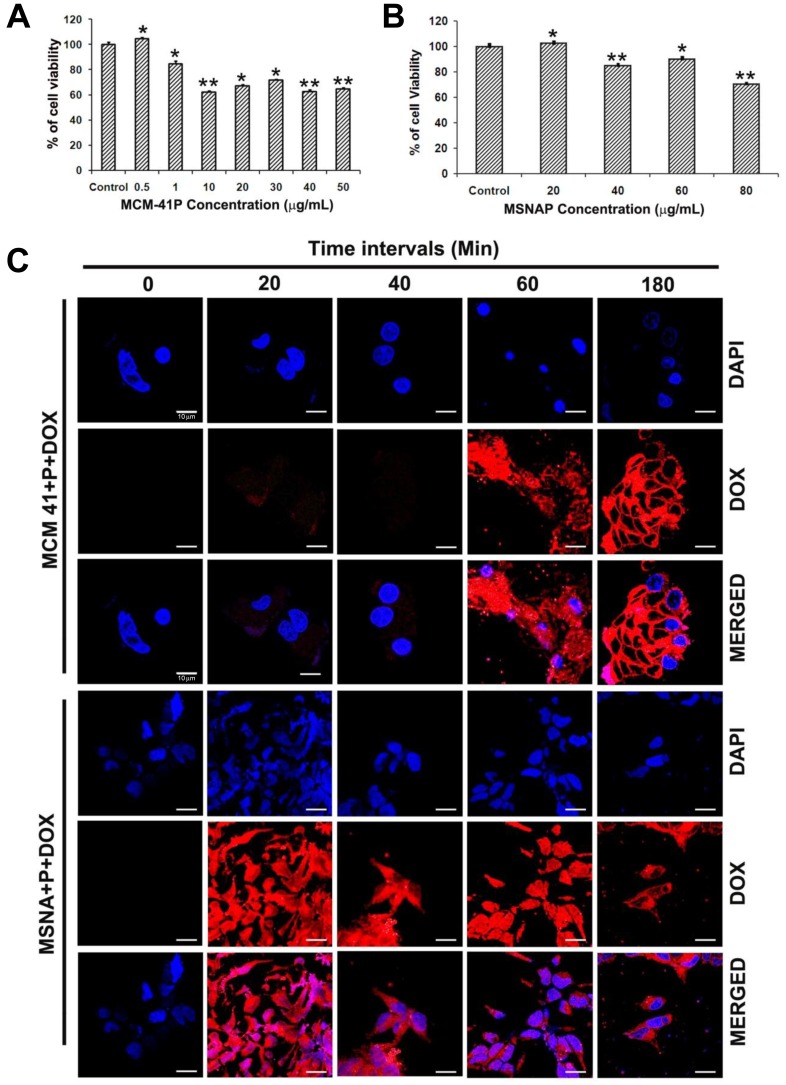
Toxicity and accumulation time of MCM-41P and MSNAP in MCF-7 cells (**A**) Graph representing cell viability percentage of MCF-7 cells in presence of increasing concentration of MCM-41P from 500 ng/mL to 50 µg/mL for 24 h. (**B**) Bar diagram representing MCF-7 viability on treatment with MSNAP from 20–100 µg/mL for 24 h. (**C**) Confocal images of MCF-7 cells with MCM-41P and MSNAP coated with DOX (red) with its corresponding DAPI (blue) staining at 0, 20, 40, 60, 180 min. and its corresponding images (DOX and DAPI) merged in MCF-7 cells. *n* = 3, *p* < 0.05 value was obtained in the treated groups compared to the control.^**^ indicates *p* value of less than or equal to 0.01 compared to control. ^*^ indicates a *p* value less than 0.05.

Toxicity of nanoparticles against MCF-7 cells assessed with WST assay indicates LD_50_ of MCM-41P was 10 µg/mL (Figure 2A) however; the LD_50_ of MSNAP was 80 µg/mL (Figure 2B) after 24 h. MSNAP was non-toxic until 20 µg/mL and even at 60 µg/mL, MSNAP induced 10% of cell death. Hence a non-toxic concentration of 30 µg/mL was used in further experiments.

Time point accumulation study (Figure 2C), suggest MCF-7 cells uptake DOX-MCM-41P from 60 min. and a maximum saturation of nanoparticles was obtained at 180 min. Whereas DOX-MSNAP uptake by MCF-7 cells was observed from 20 min. and a maximum uptake was observed at 180 min. These indicate that the rapid cellular accumulation of MSNAP in MCF-7 cells than MCM-41P [23].

### TEM based understanding of non-toxic nature exhibited by MSNAP

**Figure 3:**
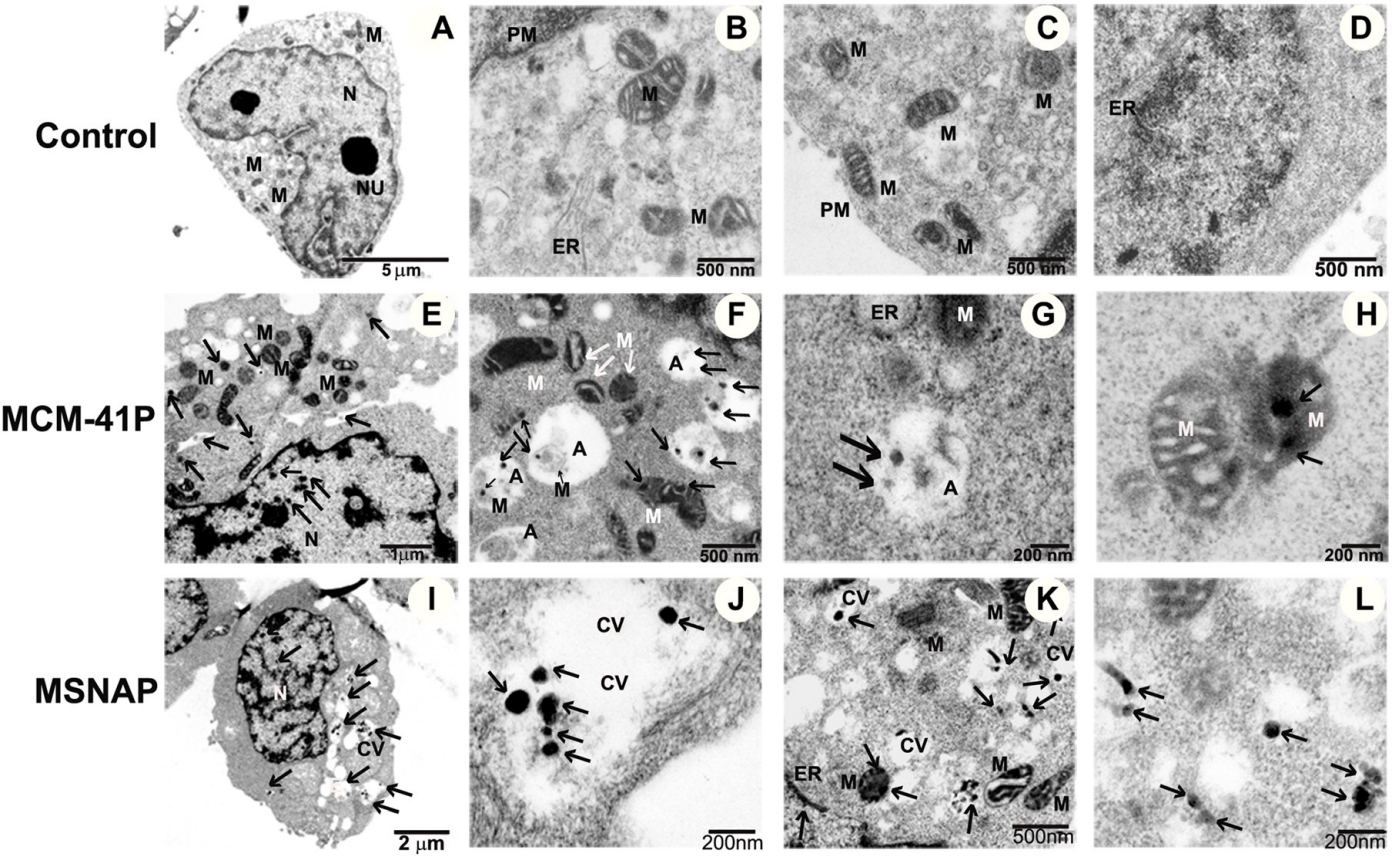
Subcellular localization of MCM-41 and MSNAP (**A**–**D**) 5 µm, 500 nm and 200 nm TEM images of control MCF-7 cells with nucleus (N), endoplasmic reticulum (ER) mitochondria (M), Golgi (G), plasma membrane (PM). Images of MCM-41P treated MCF-7 cells (**E**) 2 µm image of cell, (**F**) 500 nm image indicating MCM-41P in autophagosome (A) (**G**) 200 nm image indicating MCM-41P treated MCF-7 cells with bulged ER, (**H**) MCM-41P localized in mitochondria. Images of MSNAP treated MCF-7 cell (**I**) whole cell, (**J**) MSNAP in cytoplasmic vesicles (CV) (**K**) 500 nm image of MSNAP localised in mitochondria and (**L**) 200 nm image with MSNAP present in cytoplasm. Black arrows indicate the presence of nanoparticle.

Intracellular localization of MCM-41P and MSNAP was analyzed using a transmission electron microscope in MCF-7 cells. Cells treated with MCM-41P and MSNAP showed an increased cellular vacuolization compared to control cells (Figure 3A–3D). It was observed that a significant number of MCM-41P and MSNAP particles were localized in vacuoles (Figure 3F, 3G and 3J). MCM-41P primarily localized in mitochondria (Figure 3H) and also in autophagosomes along with the degrading mitochondria (Figure 3F). Whereas MSNAP was not accumulated (Figure 3I–3K) in any organelle and it was mostly distributed in cytoplasm and cytoplasmic vesicles. Toxicity of MCM-41P was also confirmed by the formation of dilated ER in MCF-7 cells (Figure 3G), which was not observed in control cells. However, in MSNAP treated cells no autophagosomes were observed and MSNAP was mostly found in cytoplasm (Figure 3L) and also in cytoplasmic vesicles (Figure 3J).

### MSNAP efficiently delivers curcumin to the MCF-7 cells resultantly induce apoptosis

MSNAPs drug delivery capacity was assessed with CUR-MSNAP induced cell death. MCF-7 cells treated with CUR-MSNAP were subjected to viability assay and flow cytometry to analyze the percentage of cell death. Further to confirm MSNAP released curcumin inside the MCF-7 cells, intracellular curcumin concentration was determined using nanodrop.

**Figure 4:**
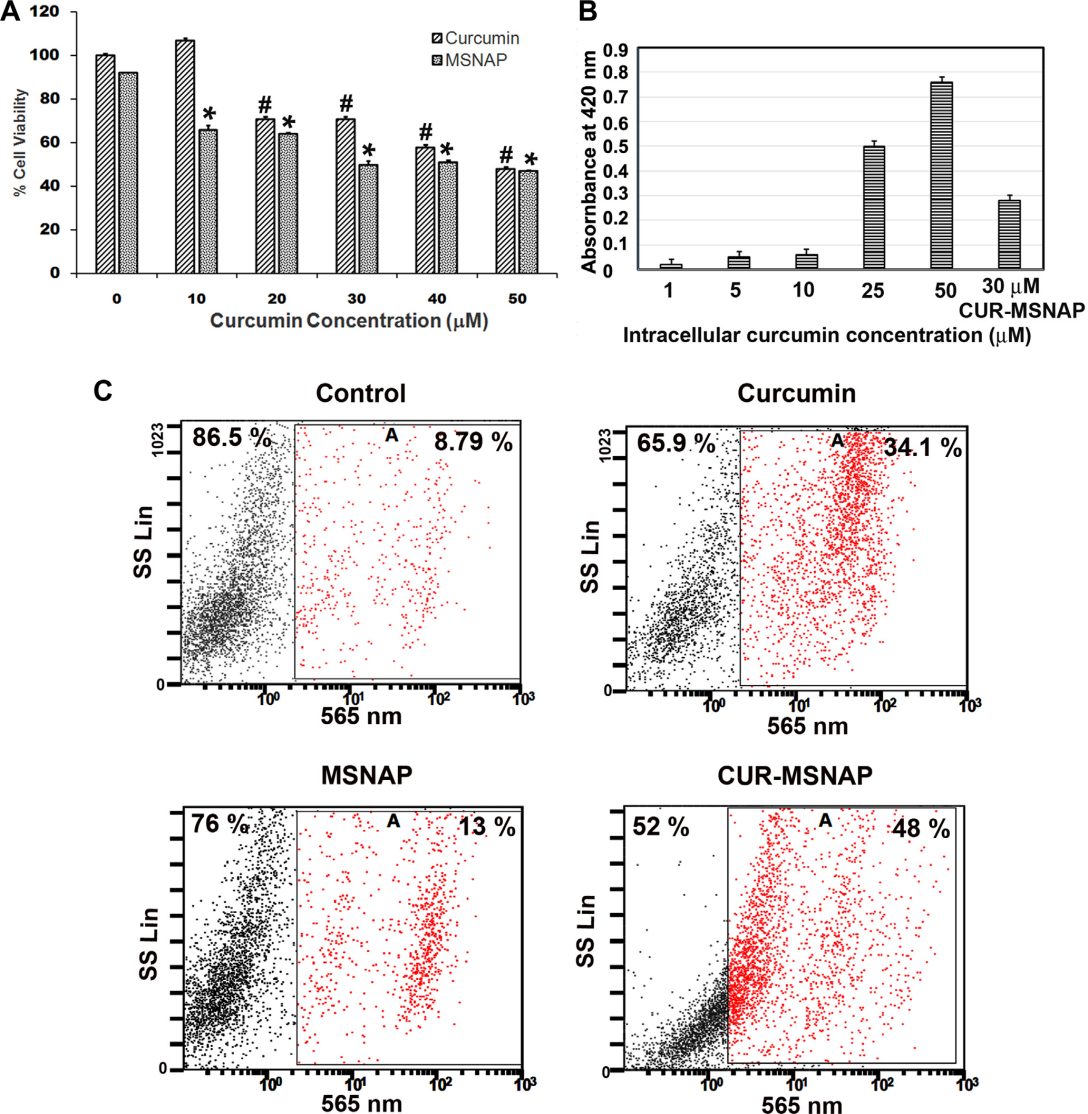
Effects of curcumin released from MSNAP (**A**) Bar diagram of MCF-7 cells viability on treatment with 0–50 µM curcumin and MSNAP (30 µg/mL) loaded with 0–50 µM of curcumin. (**B**) Graph indicating the intracellular curcumin concentration released from MSNAP at 72 h. with standard curcumin. (**C**) FACS data of live and dead cell quantification of MCF-7 cells with curcumin, MSNAP and CUR-MSNAP. *n* = 3, a significant *p* value of 0.05 was obtained comparing control and treated samples. ^*^indicates *P* < 0.05 significance of CUR-MSNAP compared to their respective control. ^#^indicates *P* < 0.05 significance of curcumin treated cells compared to the untreated control.

IC_50_ concentration of unbound curcumin and CUR-MSNAP was determined using cell viability assay. Unbound curcumin-induced 50% cell death at 50 µM concentration but CUR-MSNAP was able to induce the similar cell death at 30 µM (loaded concentration) as shown in Figure 4A. Intracellular curcumin concentration was estimated using cellular extracts from cells treated with curcumin and CUR-MSNAP. The absorption of curcumin in the cellular extract was measured at 420 nm and which showed an effective concentration of 14 µM (Figure 4B). Unbound curcumin induces cell death at 50 µM while curcumin released by MSNAP achieves a similar cell death at a lower intracellular concentration. Similarly, analysis of cell death using propidium iodide followed by flow cytometric analysis showed that the cells treated with unbound curcumin had 34% cell death while CUR-MSNAP treated cells exhibited 48% cell death (Figure 4C).

Our viability assay and FACS data suggest that 30 µM curcumin loaded on MSNAP (14 µM effective concentration) was able to induce a similar percentage of cell death as that of 50 µM of unbound curcumin. Higher intracellular accumulation and sustained drug release from MSNAP induced cell death at lower curcumin concentration compared to extracellularly administered curcumin.

### CUR-MSNAP induce apoptosis by targeting mitochondria

Further, in order to understand the mechanism of MSNAP released curcumin-mediated apoptosis, changes in activation of signalling proteins regulating apoptosis was studied in MCF-7 cells. We thus analyzed the change in expression of the protein which is involved in ER homeostasis, apoptosis and cell survival on curcumin, and CUR-MSNAP treatment.

**Figure 5:**
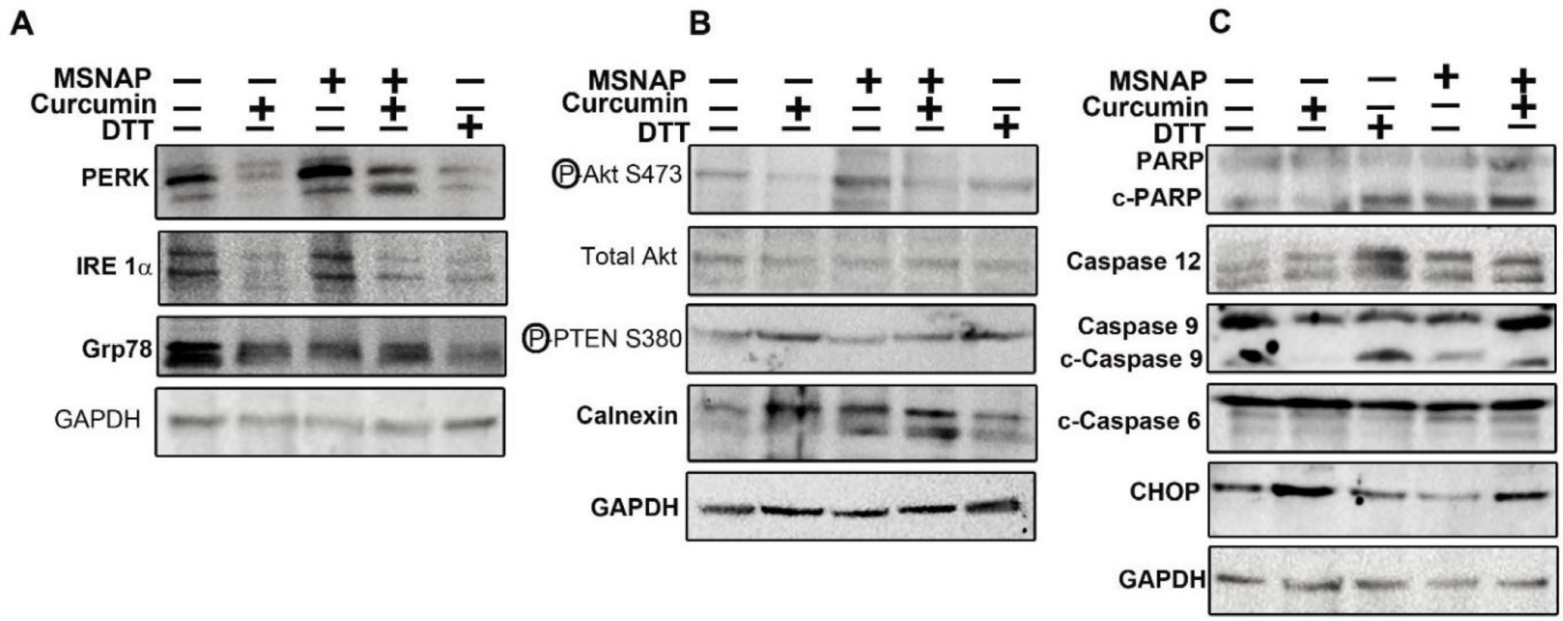
Signalling induced by intracellular released curcumin from MSNAP (**A**) Images of blot probed for PERK, IRE1α, GRP78 (**B**) Panel indicating western blots of pAkt, TAkt, PTEN and calnexin (**C**) Blots of proteins involved in apoptosis.

CUR-MSNAP and unbound curcumin increased the expression of CHOP, cleaved PARP, caspase 9, cleaved caspase 9, caspase 12, calnexin and PTEN (as shown in Figure 5B–5C). Additionally, expression of pAkt, IRE1α, PERK, and GRP 78 proteins were markedly downregulated on treatment with curcumin and CUR-MSNAP (Figure 5A–5B). Calnexin, an ER protein, expression was upregulated to two folds of curcumin and CUR-MSNAP treatment (Figure 5B).

Unbound curcumin and CUR-MSNAP downregulated Akt phosphorylation at Ser 473 however total Akt level was not altered on their treatment. Treatment of cells with curcumin and CUR-MSNAP enhanced phospho PTEN (ser 380) expression by 1.75 fold compared to the untreated cells (Figure 5B). Immunoblot study with the cleaved PARP (C-PARP) and caspase 12 showed no significant variation in their expression upon treatment with unbound-curcumin and untreated cells (Figure 5C). However, C-PARP and caspase 12 expressions were increased two folds upon CUR-MSNAP treatment as compared to the control (Figure 5C). Caspase 9 and cleaved caspase 9 expressions were also elevated on CUR-MSNAP treatment and a fourfold increase in expression of cleaved caspase 9 was observed. Unbound curcumin and CUR-MSNAP treatment elevated expression of CHOP to 1.5 fold and 1.3 fold respectively compared to the control cells. But no significant changes in caspase 6 and c-caspase 6 was observed in MCF-7 cells treated with CUR-MSNAP, when compared to the control cells.

**Figure 6:**
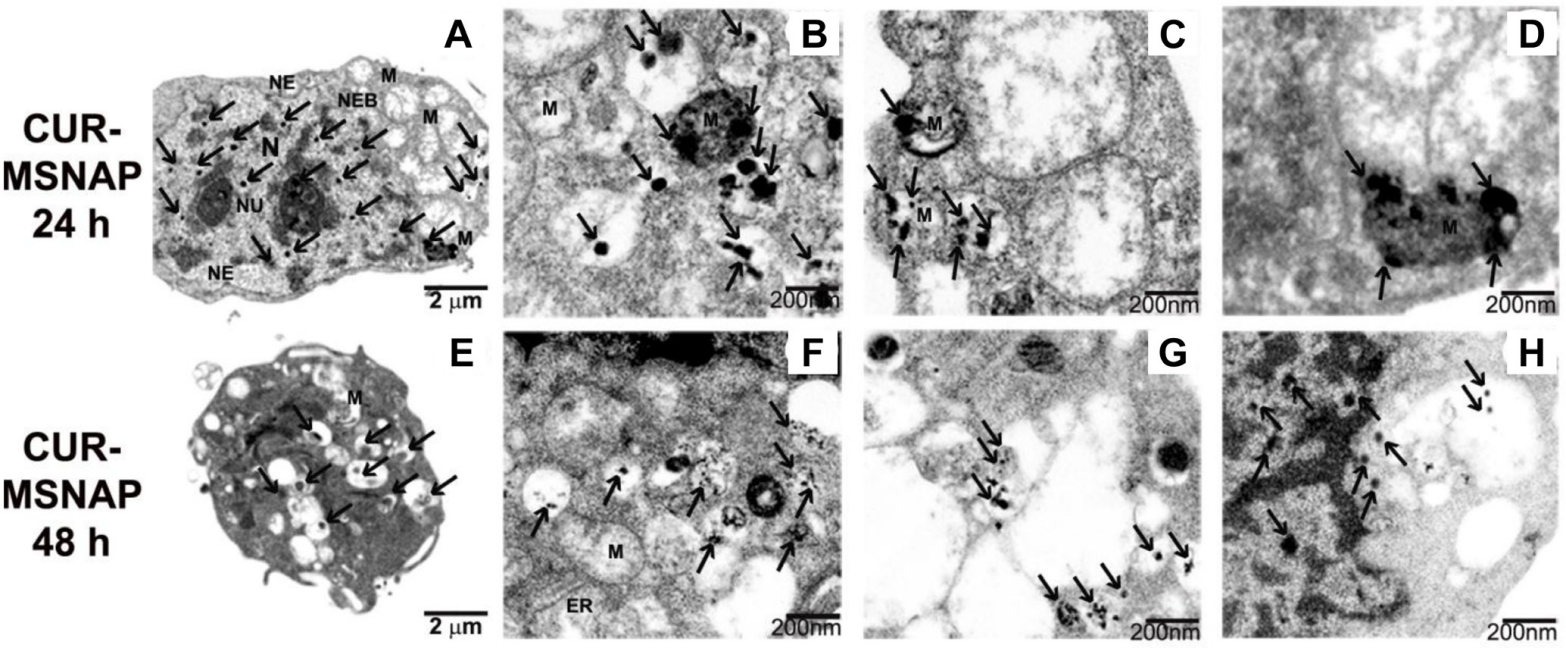
Apoptosis induced by CUR-MSNAP TEM images indicating MCF-7 cells treated with CUR-MSNAP for 24 h. (**A**) whole cell with (N) nucleus, (Nu) Nucleolus, (MS) Swollen Mitochondria, (NE) Nuclear envelop, black arrows indicating MSNAP, (**B**–**D**) 200 nm TEM image with swollen mitochondria. TEM images of CUR-MSNAP treated MCF-7 cells for 48 h. (**E**) whole cell, (**F**), (**G**) 200 nm scale image with fully swollen mitochondria (**H**) 200 nm scale image with disrupted nuclear membrane. Black arrows indicate CUR-MSNAP.

Further CUR-MSNAPs induction of apoptosis was studied by understanding the ultrastructural changes of MCF-7 cells. The MCF-7 cells were treated with 30 µM curcumin loaded MSNAP for 24 h and 48 h. Bio-TEM images of these samples indicated that CUR-MSNAP treated cells at 24 h and 48 h had distinct morphological changes observed in mitochondria and nucleus as compared to cells with MSNAP alone and control cells. MSNAP (Figure 3L) localizes primarily in the cytoplasm whereas curcumin loaded MSNAPs are mostly distributed in nucleus and mitochondria. CUR- MSNAP treated cells at 24 h (Figure 6C, 6D) showed swollen mitochondria with cristae and disrupted nuclear membrane. Additionally, cells treated with CUR-MSNAP at 48 h (Figure 6G, 6H) showed cells with swollen mitochondria, damaged plasma membrane and apoptotic bodies. Figure 6C, suggest that CUR-MSNAP treated cells at 24 h exhibit swollen mitochondria with the vesicular inner membrane. However, 48 h after incubation (Figure 6G) lost vesicular inner membrane and a swollen outer mitochondrial membrane was observed.

### DOX-MSNAP inducing cell death in MCF-7R cells

The ability of MSNAP in delivering drug to resistant cell lines was studied in DOX-resistant breast cancer cell lines. MCF-7R cells treated with MSNA-DOX-induced cell death at a lesser concentration of DOX compared to free DOX.

**Figure 7:**
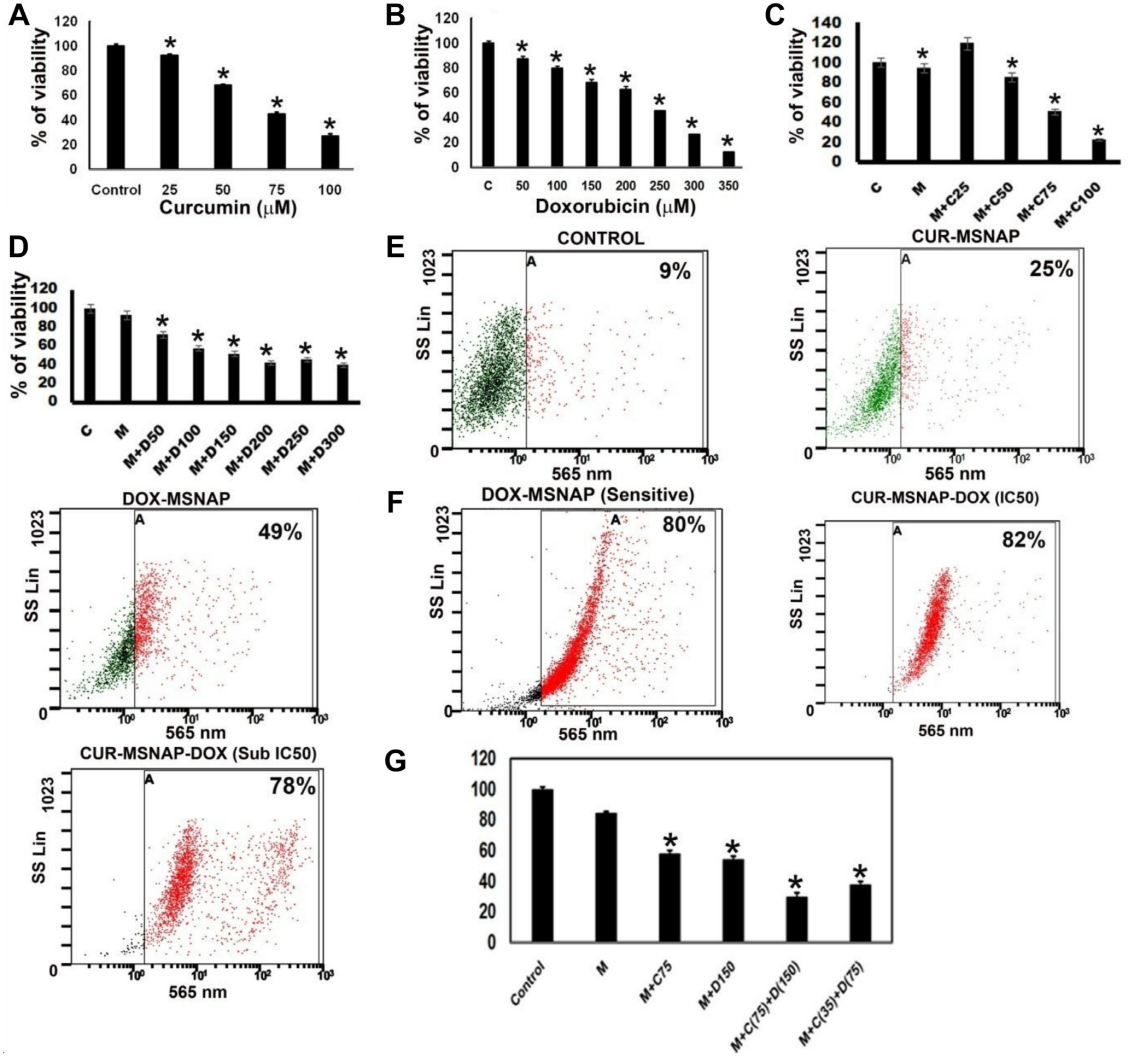
Effect of CUR and DOX loaded MSNAP on MCF-7R Graph of viability assay result with different concentrations of (**A**) CUR (**B**) DOX (**C**) CUR-MSNAP (M+C) (**D**) DOX-MSNAP (M+D) against MCF-7R. (**E**) FACS data with SSC vs FL3 revealing cell death percentage of Control, CUR-MSNAP (M+C), DOX-MSNAP (M+D), CUR (75 µM)-DOX (150 µg/mL)-MSNAP (M+C+D), CUR (35 µM)-DOX (75 µg/mL), (**F**) FACS data representing MSNA-DOX (150 µg/mL) induced percentage of cell death in MCF-7 sensitive cells (**G**) representative graph with viability assay data in MCF-7R. *n* = 3, ^*^indicates significance of *P* < 0.05 compared to their respective control.

Viability assay indicates that (Figure 7B) IC_50_ concentration of unbound DOX was 250 µg/mL found in resistant cells whereas DOX-MSNAP induced the same effect at 150 µg/mL (Figure 7D). However, there were no significant differences observed in unbound curcumin and CUR-MSNAP in their inhibitory concentration. IC_50_ of CUR-MSNAP and free curcumin in MCF-7R was 75 µM (Figure 7A–7C). The similar effect in cell death was observed in flow cytometric analysis. However, when MSNAP co-loaded with curcumin and doxorubicin (CUR-DOX-MSNAP), an increased percentage of cell death were observed (Figure 7G). Flowcytometric data indicates that DOX-MSNAP at 150 µg/mL induces 49% (Figure 7E) of cell death and CUR-MSNAP induces 25% cell death in MCF-7R cells. CUR (75 µM)-DOX (150 µg/mL)-MSNAP showed enhanced 82% of cell death (Figure 7E). Enhanced cell death of 78% was observed at half the IC_50_ concentration of CUR (37.5 µM) and DOX (75 µg/mL) loaded on MSNAP (Figure 7E) in MCF-7R cells. Similar percentage of cell death was also obtained with viability assay in MCF-7R cells (Figure 7G) with CUR-DOX-MSNAP. IC_50_ (curcumin 50 µM and Doxorubicin 150 µg/mL) concentration of curcumin and doxorubicin loaded on MSNAP induced 80% of cell death and at sub-IC_50_concentrations (Cur 25 µM and DOX 75 µg/mL), induced 78% of cell death. IC_50_ of DOX against MCF-7 cells was 100 µg/mL (data not shown), whereas IC_50_ of DOX against MCF-7R was 250 µg/mL. But DOX loaded MSNAP induced 50% of cell death at concentration of 150 µg/mL in MCF-7R. However, at the same concentration of DOX-MSNAP (150 µg/mL) induced nearly 80% of cell death in sensitive MCF-7 cells.

### Curcumin docking studies

Curcumin docking with apoptosis associated proteins was tabulated (Table 1).

**Table 1:** Parameters of curcumin docking with proteins

	Caspase 3	IRE 1 α	PARP	PERK	PTEN	Caspase 9	Akt1
Binding energy	−4.86	−6.99	−4.92	−4.39	−6.59	−4.16	−4.84
Ligand efficacy	−0.18	−0.26	−0.18	−0.16	−0.24	−0.15	−0.18
Inhibitory constant	275.8 µM	7.53 µM	245.58 µM	609.67 µM	14.8 µM	894.81 µM	282.16 µM
Intermolecular energy	−6.98	−8.64	−6.84	−5.68	−8.51	−5.68	−6.55
Vdw_desol_energy	−6.74	−8.42	−6.48	−5.31	−8.12	−5.52	−6.23
Electrostatic energy	−0.62	−0.22	−0.83	−0.37	−0.39	−0.16	−0.31
No. of H bonds	2	1	3	2	4	1	3

**Figure 8:**
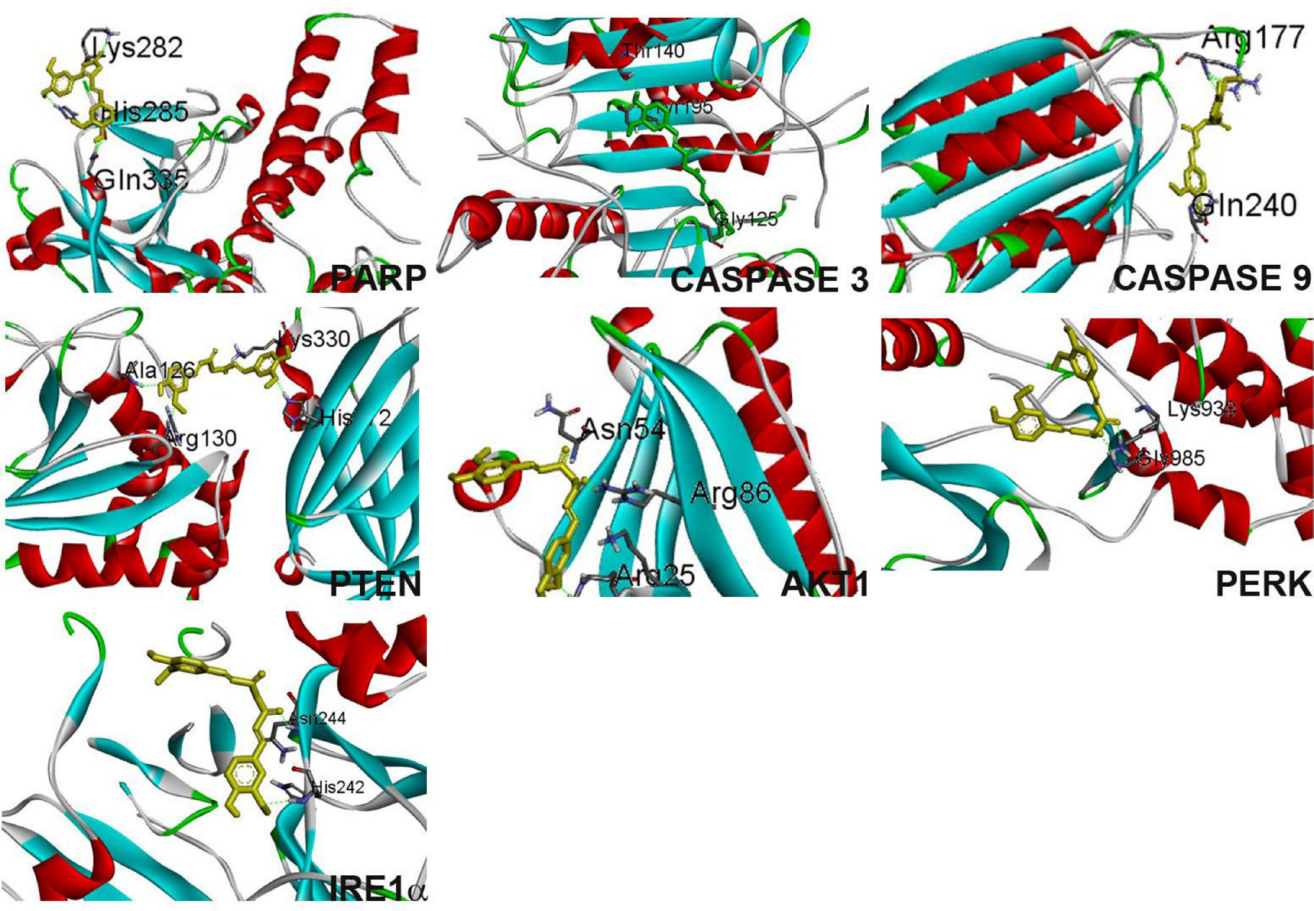
Curcumin binding confirmation with proteins PARP, caspase 3, caspase 9, PTEN, Akt1, PERK, IRE1α

Curcumin interacts with caspase-3 (Figure 8) by forming 2 hydrogen bonds between curcumin phenolic ring and tyrosine 195 and also with glycine 125. Additionally C = O group of curcumin interaction with arginine 164 through pi-pi bonding. IRE 1α binds with curcumin through a hydrogen bond with asparagine 244. Other than H-bonding, histidine 242 of the protein interacts with curcumin phenolic ring.

Curcumin forms 3 hydrogen bonds with PARP’s lysine 282, two hydrogen bonds were formed from phenolic ring with histidine 285 and glycine 335. Curcumin bends to interact (Figure 8) with PARP to form a stable confirmation with binding energy -4.92. Curcumin binds with PERK by forming two hydrogen bonds with glycine 985 and lysine 938. Curcumin interacts with PTEN with the least binding energy -6.59, 4 hydrogen bond, phenolic ring from curcumin forms bond with alanine 126, arginine 130, histidine 272 and C = O group interacts with lysine 330 with a slight bend confirmation.

Curcumin interacts with Akt1 through 3 hydrogen bond formation with arginine 25, arginine 86 and asparagine 86 with a slight bend confirmation. Curcumin interacts with caspase 9 through two hydrogen bond with arginine 177, and glycine 240.

## DISCUSSION

Exploration of new nanoparticles for better efficiency and reduced toxicity resulted in the development of non-spherical MSNAP. Synthesis of MSNs is usually achieved by two methods (a) simultaneous grafting of surfactant micelles along with silica precursor, and (b) the silica precursor is allowed to accumulate over the pre-formed surfactant micelle [24, 25]. MCM-41 was synthesized by former method [26] whereas MSNA was prepared by the latter method in midst of acid hydrolysis and condensation. Structural analysis of MSNAP with SEM revealed they were mostly in discoid shaped. Though discoid shaped is the predominant form, very few gyroids were also observed [27]. Reducing the micelle formation time and PEI coating on MSNA resulted in a non-spherical shape and smaller sized particles compared to the earlier report [5, 27]. Drug loading studies reveal MSNAP binds to curcumin 80% more than MSNA. Multivalent amino groups of PEI adsorbs the C = O and –OH groups of curcumin electrostatically, [28] which accounts for MSNAP higher drug loading. PEI coating not only enhances the efficient transfection to cells [29] but also enhances drug adsorption on the nanoparticle [30].

Curcumin release studies indicated that MSNAP released curcumin in a sustained manner, CUR-MSNAP has enhanced drug releasing capacity as compared with CUR-MCM-41P. As bigger pore sized nanostructures release drug efficiently [31], MSNAP released curcumin better due to its bigger pore size compared to MCM-41P [23]. Electrostatic interactions between the functional groups of curcumin and PEI form a zwitterionic complex. This complex is converted to protonated amines and free isomer of curcumin in water or buffer at neutral pH [31]. The gradual PEI protonation is directly proportional to the amount of drug released from PEI coated MSN. Acidic pH aided higher percentage of protonation (45%) than the neutral pH (20%) [32]. Our drug release studies data from MCM-41P showed 58 nM curcumin was released at pH 7.4 at 72 h. indicating the sustained release [33]. Thus, neutral pH played a vital role in the sustained release of curcumin from CUR-MSNAP.

Interaction of MSNAP with MCF-7 cells suggests its non-toxic nature. MCM-41P (Figure 2A) was non-toxic till 500 ng/mL and MSNAP was non-toxic till 20 µg/mL (Figure 2B). Toxicity was proved by ultra-structural changes in MCM-41P challenged cells (Figure 3F, 3G –3H). Autophagy is the cellular process to eliminate the toxins and pathogens from the mammalian cells [34]. Earlier reports with silica particles have also indicated the formation of auto-phagosome in MRC-5 cells [35]. Previous reports states in cancer cells, nanoparticles were taken up through plasma membrane and were accumulated either in mitochondria or in lysosomes. These stable solid particles cause leakage of lysosomal and mitochondrial membrane leading to cell death. The possibility of MCF-7 cells undergoing autophagocytosis could be attributed to the toxic nature of MCM-41P [36]. Whereas, MSNAP’s interaction with MCF-7 cells was non-toxic as most of the nanoparticles accumulate in cytoplasmic vesicles (Figure 3J).Nanoparticles with different shapes has different contact angle with plasma membrane. This variation in contact angle leads to the difference in particle uptake and its localisation. Non-spherical MSNAP might have generated different contact angle with MCF-7 cell membrane which could accounted for its localisation in cytoplasmic vesicle and its non-toxic nature [37].

CUR-MSNAP mechanism of inducing apoptosis was further studied in MCF-7 cells. Curcumin has been reported to induce apoptosis by modulating proteins of ER and mitochondria in numerous cancer cells [38–40]. GRP78 the HSP chaperone is the main stress sensor of ER which controls the activity of PERK, IRE1α, and ATF6. PERK, ATF6, and IRE1α dissociate from GRP 78 under stress condition and activate the downstream signalling molecules to restore the ER homeostasis [41]. Our western blot results (Figure 5A) with downregulation of PERK, IRE1α and GRP 78 by unbound curcumin and CUR-MSNAP indicate an altered ER homeostasis. Reports indicate that phosphorylation of Akt at ser473 enhances the cell survival whereas an increase in PTEN phosphorylation at ser 380 activity decreases the cell survival [42]. Our data (Figure 5B) thus suggested that unbound curcumin and CUR-MSNAP may regulate the cell survival by modulating the phosphorylation status of Akt and PTEN in MCF-7 cells. Caspases are the link between regulations of cell death and inflammation [43]. Proteolytic cleavage of caspases amplifies the signal to induce apoptosis [44]. Caspase 12 aid in cleaving procaspase 9 which is cascadically cleaves caspase 3 [45]. Our result suggested that (Figure 5C) unbound curcumin and CUR-MSNAP apoptosis activation may be implemented through caspase 12, caspase 9 and PARP. Cleaved caspase 9 was activated more than four folds compared to free curcumin administered cells. This proves that CUR-MSNAP induced better apoptosis than free curcumin. Our result was in consistent with the previous report where poly (ethylene glycol) methyl ether-b-(poly lactic acid-co-poly (b-amino esters)) of paclitaxel-induced better apoptosis in leukemic K562 cells than free paclitaxel [46].

Apoptosis mediated through mitochondria induces alteration in the inner mitochondrial membrane convoyed with cisternae degradation. Vesicular structure of the mitochondrial inner membrane was altered which eventually leads to the loss of cisternae [47–50]. Ultrastructural images (Figure 6) suggest that CUR-MSNAP influenced cells to undergo apoptosis by remodelling the inner mitochondrial membrane from normal vesicular structure to swollen vesicular at 24 h and completely swollen mitochondria at 48 h. Interestingly, it has been reported that unbound curcumin induces apoptosis by damaging chromosome and the plasma membrane in MCF-7 cells, however, there is no report to suggest that unbound curcumin cause mitochondrial insult [51]. Lv et al. reported free curcumin-induced apoptosis in breast cancer cells by mitochondrial insult with cisternae degradation at 48 h [52]. Our TEM result (Figure 6C–6D) signifies that CUR-MSNAP induced the similar effect at 24 h. This further emphasizes the advantage of MSNAP in curcumin delivery to cancer cells. The faster intracellular accumulation of the MSNAP could have contributed to earlier cisternae degradation by CUR-MSNAP than the free curcumin. Caspases are involved in mitochondria-mediated apoptosis [53, 54]. CUR-MSNAP treatment resulted in mitochondrial disruption might be the cause for increased expression of cleaved caspase 9 and caspase 12 as compared to unbound curcumin treatment.

In MCF-7R cells, though curcumin (Figure 7E, 7F, 7G) alone did not significantly affect the cell death, curcumin in combination with DOX loading on MSNAP, enhanced the percentage of cell death. Similar to other previous reports, nano delivery of DOX induces 50% of cell death at a lower concentration of drug than the native drug [55]. Our results with the effect of MSNAP loaded curcumin and doxorubicin on MCF-7 and MCF-7R suggest, MCF-7R could be sensitised by nanodrug. Further, resistance in MCF-7R was confirmed by its sensitisation to DOX. IC_50_ of DOX in MCF-7R was obtained at 250 µg/mL (Figure 7B) whereas in sensitive MCF-7 cells, IC_50_ concentration of DOX was 100 µg/mL (data not shown). Additionally, DOX resistance was also cross checked with DOX-MSNAP in MCF-7 cells. The IC_50_ concentration of DOX in MCF-7R (DOX-MSNAP 150 µg/mL) was challenged in sensitive cells which yielded in nearly 80% of cell death (Figure 7F). Drug resistance in cancer cells could be due to increased drug metabolism, drug efflux, drug inactivation or modification of drug targets [56]. Nanocarrier shaves the ability to accumulate in tumour tissue either passively or actively [57]. As most of the nanocarriers were taken up through endocytosis mediated pathway which can be bypass the drug efflux mechanism [58]. In DOX resistant uterine sarcoma tumour cell line MES-SA/Dx-5, liposome coated copper MSN with DOX induce apoptosis at a lower concentration compared to the pure drug [59]. Similarly, our preliminary finding suggests that CUR-DOX-MSNAP induced cell death in MCF-7R cells with lesser drug concentration.

## MATERIALS AND METHODS

### Synthesis of non-spherical nanoparticle

‘Origami’ method was adopted for the synthesis of non-spherical MSN (MSNA) with slight modification [27]. In brief, H_2_O, HCl, formamide, CTAB (Cetyltrimethylammonium bromide) were mixed in a molar ratio of 100:7.8:10.2:0.11 and magnetically stirred at 600 rpm for 40 h. at room temperature. Silica formation was initiated by adding 0.3 mL TEOS drop wise to this mixture and incubated further for 18 h. The template was removed by refluxing in HCl and methanol (1:20) overnight. The obtained nanostructures were coated with 0.3% of 10 kDa PEI (Alfa Aesar) [5]. MCM-41 was synthesized and coated with PEI as described earlier [33].

### Characterization of MSNAP

Structural analysis of MSNAP was carried out with TEM and SEM. MSNAP was dried on carbon paper for SEM (Evo18 Zeiss Munich, Germany), which executes at 20 KV and with Energy Dispersive X-ray (EDX) (Bruker, Madison, WI, USA). MSNAP was dried on carbon grids for High-resolution transmission electron microscope (HRTEM, T12 tecnai, Hillsboro, Oregon USA) HT650 ES1000W t 120 kV. The pore size of these nanoparticles was measured by image J software of HRTEM.

### Drug loading and release studies

10 mg of synthesized MSNAP was suspended separately in 5 mM curcumin (Alfa Aesar) in ethanol for 24 h. in an orbital shaker. The unbound free curcumin was removed after 24 h. and its absorbance at 420 nm was compared with 0 h. in nanodrop (Biospec Nano, Shimadzu). Percentage of curcumin loaded in both nanostructure was determined by the formula (Abs at 0 h- Abs at 24 h/ Abs at 0 h)*100.

The concentration of curcumin released from CUR-MSNAP (30 µM curcumin loaded with 30 mg/mL MSNAP) as determined in phosphate buffer saline (PBS) at pH 7.4. Initially, 4 mg/mL of both the MSN loaded with curcumin was immersed in PBS separately till 96 h Curcumin released in PBS was analyzed at every 12 h at 420 nm in nanodrop. The concentration of curcumin released was calculated by referring to the standard curcumin graph.

### Cell culture

Breast adenocarcinoma (MCF-7) cells were cultured in IMDM (Gibco/Life Technologies, Gaithersburg, MD, USA) medium with 10% FBS (Gibco BRL) in 5% CO_2_ incubator at 37°C. Serum-free media was used in all the experiments involved with nanoparticle.

### Development of doxorubicin resistant MCF-7 cells (MCF-7R)

Doxorubicin (DOX) (Doxotero, Hetero HC, Hyderabad, India) resistant cells were developed by adapting the MCF-7 cells to increasing concentration of doxorubicin from 500 ng/mL to 33 µg/mL [60] (Clinically relevant resistant DOX concentration in patient’s plasma). Initially, MCF-7 cells were exposed to 500 nM of DOX for 48 h. and retrieved in fresh media until the plate reached confluence. The same procedure was repeated with the higher concentrations from 1 µM to 33 µM.

### Toxicity assays

#### Viability assay

Cell death induced by MSN, was assessed with water-soluble tetrazolium-1 (WST-1) reagent (Roche, Germany GmbH). Briefly, 10,000 cells were seeded in 96 well plate (Greiner, Bio-One, Ireland) and allowed to adhere overnight. MSNAP and MCM-41P of concentration from 0.5 µg/mL to 100 µg/mL were added to the plate in triplicates and incubated for 24 h. followed by addition of 5 µL WST-1. The plate was read at 450 nm in a microplate reader (Biotek, Model FLx800, Vermont, USA). Percentage of live cells was calculated from formula (OD of sample/OD of control) X100.

### Accumulation of MSNs in MCF-7 cells

Accumulation of nanoparticles in MCF-7 cells was analyzed by confocal laser scanning microscopy (CLSM), (LSM 500, Zeiss, Munich, Germany). Cells were grown on coverslips till they attain 60% confluence. 5 nM DOX (Doxotero, Hetero HC, Hyderabad, India) loaded silica nanostructures (DOX-MSNAP) were incubated with MCF-7 cells for different time intervals such as 0, 20, 40, 60, 120 and 180 min. Then the coverslips were fixed with 4% paraformaldehyde followed by DAPI staining. Cells were imaged with CLSM with excitation at 405 nm and emission from 580 to 620 nm [33].

### Bio-TEM studies for subcellular localization of MSNs in MCF-7 cells

The non-toxic concentrations of MCM-41P and MSNAP were incubated for 72 h. and 30 µM of CUR-MSNAP was incubated for 24 and 48 h. in MCF-7 cells. After incubation cells were harvested and fixed in a fixative mixture of 2.5% glutaraldehyde, 2% sucrose and complete media for 12 h. Then the fixed cells were stained with 1% osmium tetroxide. Followed by dehydration in a series of ethanol from 70%–100%. The fixed cells were added with 100% propylene oxide then gradually transferred to 100% epoxy resin (TAAB, England) by decreasing the percentage of propylene oxide. Finally, cells were kept in pure resin for 5 h. and embedded in a freshly prepared resin at 50°C for 48 h. Resin embedded samples were made to 80–100 nm thin sections with ultra-microtome. These sections were counterstained with uranyl acetate and lead citrate and imaged with transmission electron microscope (Tecnai G^2^, Hillsboro, Oregon, USA) at 80 KV.

### Evaluation of CUR-MSNAP induced apoptosis in MCF-7 cells

#### Viability assay

IC_50_ value of CUR-MSNAP in MCF-7 cells was determined by WST-1 assay as mentioned earlier. Cells were treated with different concentration of curcumin (5–50 µM) loaded on non-toxic concentrations of 30 µg/mL MSNAP. After 72 h, cell viability was measured with WST-1 reagent as described earlier [33]. Percentage of live cells was determined by the formula (Absorbance of treated cells / Absorbance of control cells)*100.

FACS analysis was used to quantify live and dead cells on curcumin, MSNAP, CUR-MSNAP treatment. Cells were grown in 12 well plate (Greiner) and treated independently with 30 µg/mL MSNAP, 50 µM curcumin, and 30 µM-CUR-MSNAP for 72 h. Cells were harvested and stained with 5 µL of 10 µg/mL propidium iodide (PI) for 10 min. The stained cells were analyzed in FACS (FC500, Beckman Coulter, Brea, CA, USA) and PI positive cells were gated in FL3. The forward scatter (FSC) and side scatter (SSC) was also analyzed simultaneously.

### Measurement of intracellular curcumin released from nanoparticles

The concentration of curcumin released from CUR-MSNAP in MCF-7 cells was determined by nanodrop. Briefly, 20,000 cells were grown in 12 well plate and incubated with 30 µM CUR-MSNAP (IC_50_ value) and with standard curcumin (solubilized in ethanol) concentrations (1, 5, 10, 25, 50 µM). After 72 h, the cells were harvested and lysed with lysis buffer (Tris pH 10, 150 mM NaCl, 10% DMSO) for 30 min. and the lysate was sheared with 25 gauge needles followed by centrifugation at 12,000 rpm for 10 min. at 4°C. The supernatant was measured at 420 nm in nanodrop. The absorbance of curcumin was compared with standard curcumin graph.

### Western blot analysis

The qualitative differences of CUR-MSNAP influenced protein expression in MCF-7 cells were analyzed using Western blot. 2 × 10^6^ cells grown in 100 mm dishes (Greiner) were treated with 50 µM curcumin, 30 µg/mL MSNAP, 30 µM CUR-MSNAP, and 15 µM DTT for 72 h. DTT was used as a positive control for UPR induction [61]. After incubation, MCF-7 cells were lysed with RIPA (Radio immunoprecipitation assay) buffer (pH 7.4) containing protease and phosphatase cocktail inhibitors (Roche, Switzerland) on ice for 20 min. and the lysates were sheared with 25 gauge needle, followed by centrifugation at 12,000 rpm for 25 min. at 4°C. Proteins were quantified with BCA reagent (Sigma-Aldrich, St. Louis, MO). Each 50 µg of proteins were loaded on SDS-PAGE and run at 110V for 2 h. The proteins were transferred to nitrocellulose membrane (Amersham Bioscience, Piscataway, NJ, USA). Anti-PERK, anti-IRE1α, anti-GRP 78, anti-calnexin, anti-phospho-Akt (Ser473), anti-total Akt, anti-phospho PTEN (ser 380), anti-PARP, anti-caspase 12, anti-caspases 9, 6, anti-CHOP, and anti-GAPDH were obtained from Cell Signalling Technology (Danvers, MA, USA). The membrane was incubated overnight with primary antibodies at 4°C followed by either anti-rabbit IgG or anti-mouse IgG HRP-linked secondary antibodies (Santa Cruz, CA, USA) for 1 h. Presence of the protein was detected with addition of lumiglo (Thermo Scientific, Rockford, IL, USA) reagent and imaged in gel documentation system (Bio-Rad, Hercules, CA, USA) with Image lab 5 software. Densitometry of respective bands was analyzed by Image J software. Expression of proteins was represented as fold change with a ratio of each protein to its loading control GAPDH.

### Analysing DOX-MSNAP induced cell death in MCF-7R cells

Viability assay was performed on MCF-7R cells with DOX, DOX-MSNAP, CUR, and CUR-MSNAP as described earlier in 96 well plate. WST reagent was used to quantify the percentage of cell death and the concentration of DOX, DOX-MSNAP, CUR, CUR-MSNAP inducing 50% cell death was predicted. Flow cytometric analysis was also used to confirm the live and dead cells population in above-mentioned conditions.

### Docking studies

Autodock 4.2 tool was used for docking curcumin with proteins caspase 3, 9, IRE 1α, PERK, PARP, Akt1, and PTEN. The grid was built for 60 × 60 × 60 in X, Y & Z directions. The binding model for each protein was analyzed with visualization tool PyMOL [62].

### Statistical analysis

All experiments were repeated in three times. Results analyzed as the mean ± standard error of the mean values. Statistical analysis of control group and treatment group was performed with student’s *t*-test (Graph pad Prism 5, Graph pad software, San Diego, CA, USA). *P* value of < 0.05 was considered statistically significant with a 95% confidence interval.

## CONCLUSIONS

In summary, non-spherical mesoporous silica nanoparticle coated with PEI was characterized for its drug delivery efficiency in MCF-7 cells. Influence of MCM-41P, MSNAP and CUR-MSNAP treated MCF-7 cells were summarized in Supplementary Figure 1. The non-spherical shape of MSNAP synthesized by origami method aids reduction in toxicity, faster intracellular accumulation, and better drug release. Drug released from MSNAP intracellularly even at lower concentration disturbs the cellular organelles and induce apoptosis. Additionally, MSNAP mediated drug delivery sensitized resistant cells at subordinate drug concentration. We report the mesoporous silica nanoparticle with non-spherical shape has to influence on cytotoxicity and drug delivery. Further *in vivo* exploitation of MSNAP will be helpful in understanding the biodistribution and bioavailability of this carrier particle.

## SUPPLEMENTARY MATERIALS FIGURE



## Abbreviations

PEI: Polyethyleneimine
MCM-41P: MCM-41 coated with PEI
MSNAP: MSNA coated with PEI
CUR-MSNAP: Curcumin loaded MSNA coated with PEI
MSN: Mesoporous silica nanoparticles
IC_50_: Inhibitory concentration (50%)
LD_50_: Lethal dosage (50%)
DOX-MSNAP/MCM-41P: Doxorubicin loaded MSNAP/MCM-41P
DDS: Drug delivery system
CUR-DOX-MSNAP: Curcumin and Doxorubicin loaded MSNAP
