# Supplementary material for: An ingenious non-spherical mesoporous silica nanoparticle cargo with curcumin induces mitochondria-mediated apoptosis in breast cancer (MCF-7) cells
Source: Oncotarget. 2019 Feb 5;10(11):1193–208. doi: 10.18632/oncotarget.26623 (PMC6383822; doi:10.18632/oncotarget.26623)
Supplement: Supplementary file 1 [file oncotarget-10-1193-s001.pdf]

An ingenious non-spherical mesoporous silica nanoparticle cargo with curcumin induces mitochondria-mediated apoptosis in breast cancer (MCF-7) cells

SUPPLEMENTARY MATERIALS

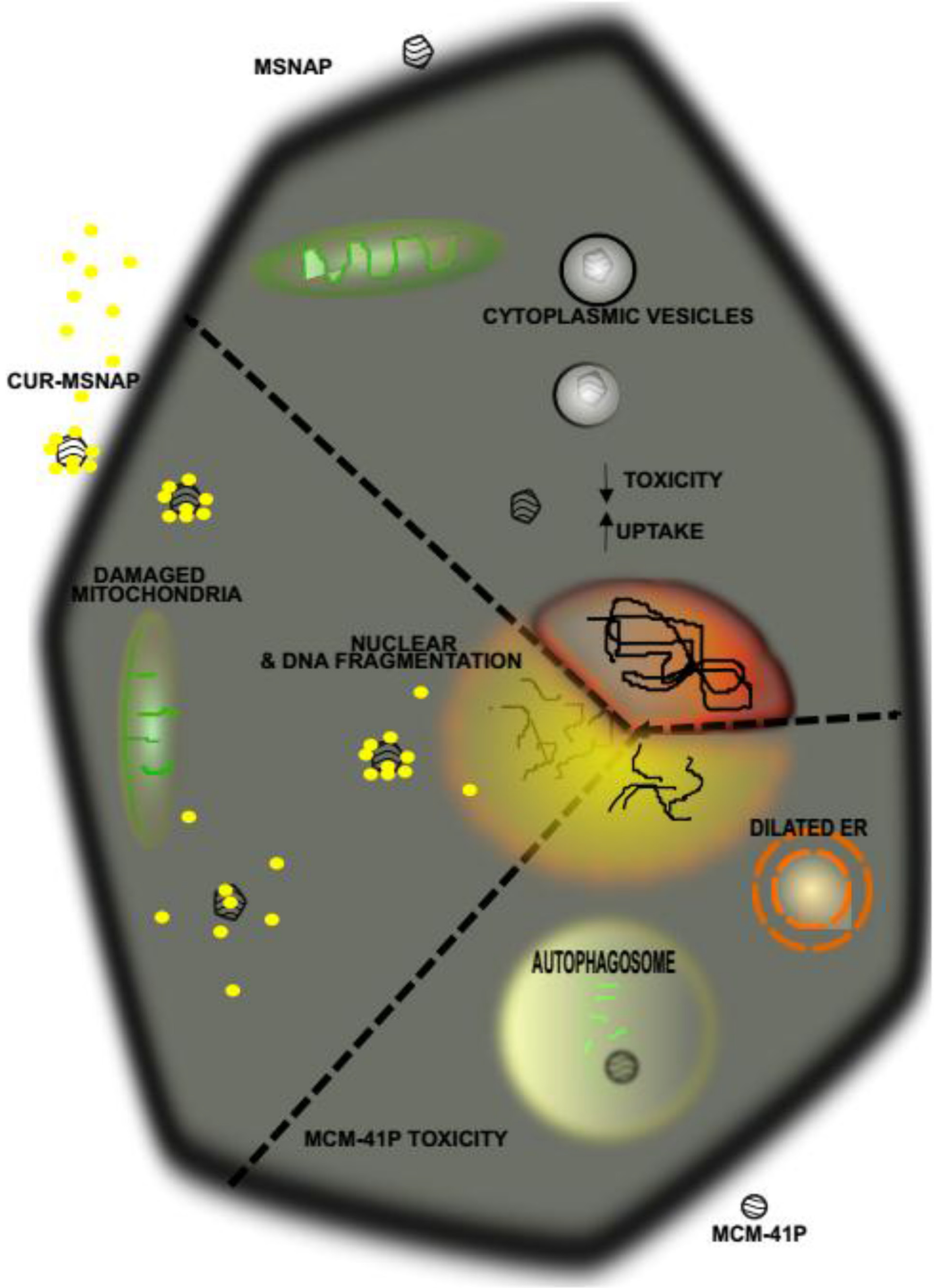

Supplementary Figure 1: Schematic overview of MCM-41P, MSNAP and CUR-MSNAP effects on MCF-7 cells. MCM-41P triggered toxic response in cells whereas MSNAP did not induce toxicity. Intracellular curcumin released from MSNAP disrupts mitochondria and nucleus eventually leading to apoptosis.
